# Investigation of the impact of brewing parameters on toxic element and rare earth element contamination in oolong tea

**DOI:** 10.3389/fnut.2025.1656046

**Published:** 2025-11-11

**Authors:** Sixuan Wu, Ziming Peng, Yijun Ou, Florence Mhungu, Yanyan Wang, Yuhua Zhang, Lan Liu, Jiangbo Lei, Lili Huang, Rongfei Peng, Zhijun Bai, Weiwei Zhang

**Affiliations:** 1 Department of Public Health and Preventive Medicine, School of Medicine, Jinan University, Guangzhou, China; 2 Department of Foodborne Disease and Food Safety Risk Surveillance, Guangzhou Center for Disease Control and Prevention (Guangzhou Health Supervision Institute), Guangzhou, China; 3 Pingxiang Center for Disease Control and Prevention, Pingxiang, China; 4 School of Economics and Statistics, Guangzhou University, Guangzhou, China; 5 Horizon Health Network, Fredericton, NB, Canada; 6 School of Public Health, Guangdong Pharmaceutical University, Guangzhou, China; 7 Department of Quality Control, Guangzhou Center for Disease Control and Prevention (Guangzhou Health Supervision Institute), Guangzhou, China; 8 Department of Physical and Chemical Inspection, Guangzhou Center for Disease Control and Prevention (Guangzhou Health Supervision Institute), Guangzhou, China

**Keywords:** oolong tea, toxic elements, REEs, brewing conditions, ICP-MS, food safety

## Abstract

**Introduction:**

With the growing consumption of oolong tea, concerns regarding the leaching of toxic elements and rare earth elements (REEs) during brewing necessitate investigation.

**Methods:**

We analyzed 108 oolong teas of diverse origins and varieties. The concentrations of six toxic elements (including Pb, Cd, Al) and fifteen REEs were measured by ICP-MS. The effects of water temperature (90°C, 100°C) and brewing time (5 seconds to 2 hours) on leaching rates were systematically examined.

**Results:**

High temperature and long brewing time significantly increased (*P*<0.05) the leaching of most elements. Tieguanyin tea contained the highest levels of Pb, Al, and REEs. Samples from Fujian province significantly exceeded safety standards for Pb and Al. Anomalously, the leaching rate of Cd was lower at 100°C than at 90°C, while the release of scandium (Sc) increased with temperature.

**Discussion:**

This study reveals that brewing conditions are critical for elemental migration. To minimize the intake of harmful substances, consumers are advised to shorten the brewing time. We also call for strengthened regulatory standards for toxic elements and REEs in tea. These findings provide a scientific basis for guiding safe tea consumption practices.

## 1 Introduction

Oolong tea, also known as Qingcha, ranks among the world’s three most famous types of tea, alongside green tea and black tea. Originating in China, it is classified as one of the six primary types of tea produced in the country (1). As the birthplace of tea, China is home to numerous tea-producing regions, with Fujian and Guangdong being particularly renowned for their oolong tea production. In 2021, China’s oolong tea production reached 287,200 tons, with domestic consumption at 227,900 tons, making it the second most-consumed type of tea (2).

As the birthplace of oolong tea, Fujian Province is renowned for producing well-known varieties such as Tieguanyin and Dahongpao. Tieguanyin from Anxi is celebrated for its unique floral aroma and sweet aftertaste (3), while Dahongpao from Wuyi Mountain is regarded as the “king of teas” due to its deep, rocky flavor and complexity (4). Guangdong Province is famous for its Phoenix monocotyledon and Daffodil teas, with Phoenix Mountain monocotyledon tea from Chaozhou distinguished by its single-plant harvesting and rich aroma, offering a diverse range of floral, fruity, and nutty notes (5). The varietal characteristics of Fujian and Guangdong oolong teas are closely tied to their regional climatic and soil conditions. This unique “terroir” influences both the quality of the tea and potentially its leaching behavior of toxic elements and REEs (6).

Relevant studies have indicated that toxic elements can accumulate in tea leaves and be released during the leaching process. Most toxic elements are capable of causing severe health damage to the human body even at relatively low concentrations (7). Among these toxic elements, lead, Cd, and Al are particularly harmful. Lead can impair the nervous system and kidneys, with children being especially vulnerable due to its potential to hinder intellectual development (8, 9). Cd is associated with kidney damage and an increased risk of cancer (10, 11), while long-term exposure to Al may contribute to neurodegenerative diseases like Alzheimer’s (12, 13). Cu, manganese, and Ni are essential trace elements for human health, but excessive intake of these can lead to adverse health effects. Excessive intake of Cu can lead to liver and kidney damage, gastrointestinal discomfort, and neurological issues (14). Manganese toxicity is associated with neurological impairments, including ataxia and cognitive dysfunction, particularly in environments with high Mn exposure (15). Ni, a common allergen, can cause dermatitis with prolonged exposure to high concentrations, and has been linked to lung and nasal cancers. Additionally, it can adversely affect cardiovascular and immune system functions (16).

REEs, which encompass lanthanide elements as well as yttrium (Y) and scandium (Sc), are contaminants of significant concern in the environment and food. Their food safety risks cannot be overlooked. During the tea planting process, REEs can enter tea plants through pathways such as soil migration, atmospheric deposition, and agricultural inputs, and subsequently accumulate in the leaves (17). Since tea is consumed directly after brewing, the bioaccessibility of REEs entering the human body via tea infusion is relatively high, posing potential dietary exposure and health risks. Relevant studies have shown that excessive intake of REEs may give rise to health hazards, including alterations in blood parameters, skin lesions, and developmental disorders in children (18–20).

Tea-drinking habits vary among individuals, with some preferring short brewing times of 30 s to 1 min, while others, particularly in certain regions, favor soaking tea leaves in large teapots for extended durations. Despite the widespread consumption and research on oolong tea, limited studies have examined the leaching of toxic elements and rare earth during brewing, highlighting the need for further investigation. Most existing research evaluates the health impact of tea by analyzing the trace element content in dry tea leaves. However, as consumers typically consume the brewed tea infusion, assessing health risks solely based on the trace element content in dry leaves may lead to inaccurate conclusions. This study addresses this gap by analyzing the concentrations of toxic elements and rare earth in both tea leaves and brewed tea infusions. It also investigates how brewing time, temperature, and repetition influence the leaching behavior of these elements in oolong tea. The findings highlight potential exposure risks to consumers and provide scientific guidance for minimizing health hazards associated with tea consumption.

## 2 Materials and methods

### 2.1 Sample collection

In 2021, a total of 108 oolong tea samples were collected from various locations in Guangzhou, including the Yuexiu, Haizhu, Liwan, Tianhe, Baiyun, Zengcheng, Huadu, Nansha, Conghua, Luogang districts, and Chaozhou City. The samples primarily included four varieties: Tieguanyin, Dahongpao, Dancong, and Narcissus. They were sourced from supermarkets, wholesale markets, retail stores, and online stores to ensure a diverse representation. The specific details are provided in Supplementary Table 1.

### 2.2 Sample analysis

The primary objective of this experiment was to measure the concentrations of toxic elements and REEs in the tea infusions. The toxic elements analyzed included lead (Pb), cadmium (Cd), aluminum (Al), copper (Cu), manganese (Mn), and nickel (Ni). Additionally, 15 REEs were quantified, such as scandium (Sc), yttrium (Y), lanthanum (La), cerium (Ce), praseodymium (Pr), and neodymium (Nd), reported collectively as total rare earth. These elements were selected based on their known health risks and prevalence in tea. The detection standards adhered to were the “National Food Safety Standard: Limits of Contaminants in Food” (GB2762-2022).

### 2.3 Major instruments and reagents

The Agilent 7,700 Series inductively coupled plasma-mass spectrometer (ICP-MS) from Agilent Technologies was used for metal element detection. The acquisition mode was set to mass spectrometry mode with the following parameters: peak shape acquisition with 3 points, 3 replicates per scan, 20 scans per replicate, and a total acquisition time of 28.860 s. Monitoring parameters and integration times/mass numbers were optimized for different metal elements: 0.99 s for Al (mass 27) and Sc (mass 45); 0.09 s for Mn (mass 55) and Ni (mass 60); and 0.30 s for all other elements (Supplementary Table 2 for detection and quantification limits).

Instrumental plasma parameters were configured as follows: RF power 1,550 W, RF matching 1.60 V, sampling depth 8.0 mm, carrier gas flow rate 0.70 L/min, peristaltic pump speed 0.20 rps, spray chamber temperature 2 C, and makeup gas flow rate 0.50 L/min. The collision cell was operated with helium gas at a flow rate of 3.8 mL/min to minimize polyatomic interferences.

#### 2.3.1 Tea sample preparation and digestion

Collected tea samples were first assigned unique identifiers. Subsequently, homogenization was performed using a food-grade grinder, and the material was passed through a 60-mesh sieve to ensure particle size uniformity. The processed samples were stored in sealed containers to prevent environmental contamination. An appropriate amount of the processed tea sample was accurately weighed and mixed with 100 μL of nitric acid (1%, v/v). Digestion was carried out using a microwave digestion system (Ethos 900, Tokyo, Japan) under conditions of 150 C, 50 bar, and 1,000 W for 1 h. After digestion, the sample was cooled to room temperature. The digestate was then transferred to a 5 mL polypropylene tube and diluted to 20 mL with ultrapure water. Finally, the metal element concentrations were analyzed by ICP-MS.

Exactly 2.000 g of tea leaves were weighed into a 200 mL beaker, and 100 mL of drinking water at a specified temperature (90°C or 100°C) was added. After steeping for 5 s, the first infusion was decanted into a 500 mL sampling bottle, while the tea residue was retained in the beaker. Then, 100 mL of fresh hot water at the same temperature was added to the residue for successive extraction periods of 5 s, 1 min, 5 min, 10 min, 30 min, and 2 h. Each infusion was collected into separate 500 mL sampling bottles. A blank control was prepared by steeping 100 mL of hot water for 5 s. For analysis, 10 mL of the second infusion was mixed with 100 μL of nitric acid (1%, v/v). Subsequent digestion and analysis steps were identical to those described for the tea leaf samples.

### 2.4 Experimental design

Following common oolong tea brewing methods reported in literature (21, 22), two brewing temperatures were selected: 90°C and 100°C. Precisely, 2.000 g of tea leaves were thoroughly mixed and brewed in 100 mL of natural mineral water at the designated temperatures. For the initial infusion, the tea was brewed for 5 s, after which the liquid was collected as the ‘first infusion,’ and the residual tea leaves remained in the beaker. The leaves were subsequently re-brewed for varying infusion durations (5 s, 1 min, 5 min, 10 min, 30 min, and 2 h). Each infusion was collected separately, with the second infusion poured into a dedicated 500 mL sampling bottle, and the residual tea leaves left in the beaker after each infusion. The trace element content in each collected infusion was analyzed using ICP-MS. For accuracy and reliability, three parallel samples were prepared and measured for each infusion time.

### 2.5 Statistical analysis

Data were organized using Excel 2021 and analyzed with SPSS Statistics 29. Due to the non-norminal distribution of data, nonparametric tests were applied for group comparisons, with a significance level set at *p* < 0.05. The content of REEs in both tea leaves and tea infusions was determined using the methods described earlier. The leaching rate was calculated using the formula:

Leaching rate = (Leached content of REEs in tea infusion/Total content of REEs in tea sample) × 100%.

## 3 Results

### 3.1 Differences in element contents among different oolong tea varieties

Among the four main oolong tea varieties (Tieguanyin, Dahongpao, Dancong, and others) tested in this study, there were significant differences in the contents of toxic elements and REEs (except for Cd, *p* < 0.001). As shown in Table 1 Tieguanyin exhibited the highest content levels for all detected elements. Its Pb content reached 0.533 ± 0.306 mg/kg, which was significantly higher than that of Dahongpao (0.491 ± 0.259 mg/kg) and Dancong (0.247 ± 0.175 mg/kg). Notably, the Al content in Tieguanyin was as high as 1549.514 ± 2736.48 mg/kg, far exceeding that of other varieties. In terms of REEs, Tieguanyin also had the highest content of total rare earth oxides (TREO). Specifically, the contents of Sc, La, and Ce in Tieguanyin were 3.704, 0.542, and 0.511 mg/kg, respectively.

**Table 1:** Comparison of leached toxic elements and REEs in different varieties of oolong tea leaves.

Element	Variety				*p-*value
	Red Robe	Dancong	Tieguanyin	Other	
Pb (mg/kg)	0.491 ± 0.259	0.247 ± 0.175	0.533 ± 0.306	0.414 ± 0.343	<0.001
Cd (mg/kg)	0.029 ± 0.012	0.028 ± 0.026	0.028 ± 0.025	0.022 ± 0.016	0.304
Al (mg/kg)	909.92 ± 318.22	453.27 ± 282.65	1549.51 ± 2736.48	754.56 ± 352.25	<0.001
Sc (mg/kg)	2.031 ± 2.031	1.144 ± 0.752	3.704 ± 2.966	1.191 ± 1.191	<0.001
Y (mg/kg)	0.039 ± 0.022	0.023 ± 0.015	0.055 ± 0.035	0.035 ± 0.035	<0.001
La (mg/kg)	0.303 ± 0.366	0.151 ± 0.183	0.542 ± 0.546	0.153 ± 0.153	<0.001
Ce (mg/kg)	0.269 ± 0.269	0.142 ± 0.142	0.511 ± 0.511	0.163 ± 0.163	<0.001
Pr (mg/kg)	0.555 ± 0.555	0.370 ± 0.370	1.020 ± 1.020	0.333 ± 0.333	<0.001
Nd (mg/kg)	0.053 ± 0.053	0.027 ± 0.027	0.101 ± 0.101	0.033 ± 0.033	<0.001
Sm (mg/kg)	0.219 ± 0.219	0.111 ± 0.111	0.414 ± 0.414	0.136 ± 0.136	<0.001
Eu (mg/kg)	0.040 ± 0.040	0.020 ± 0.020	0.073 ± 0.073	0.025 ± 0.025	<0.001
Gd (mg/kg)	0.010 ± 0.010	0.004 ± 0.004	0.016 ± 0.016	0.006 ± 0.006	<0.001
Tb (mg/kg)	0.043 ± 0.043	0.020 ± 0.020	0.075 ± 0.075	0.025 ± 0.025	<0.001
Dy (mg/kg)	0.007 ± 0.007	0.003 ± 0.003	0.012 ± 0.012	0.004 ± 0.004	<0.001
Ho (mg/kg)	0.045 ± 0.045	0.021 ± 0.021	0.076 ± 0.076	0.023 ± 0.023	<0.001
Er (mg/kg)	0.009 ± 0.009	0.005 ± 0.005	0.017 ± 0.017	0.005 ± 0.005	<0.001
Tm (mg/kg)	0.031 ± 0.031	0.016 ± 0.016	0.056 ± 0.056	0.015 ± 0.015	<0.001
Yb (mg/kg)	0.005 ± 0.005	0.003 ± 0.003	0.009 ± 0.009	0.003 ± 0.003	<0.001
Lu (mg/kg)	0.036 ± 0.036	0.021 ± 0.021	0.069 ± 0.069	0.018 ± 0.018	<0.001
TREO (mg/kg)	0.491 ± 0.259	0.247 ± 0.175	0.533 ± 0.306	0.414 ± 0.343	<0.001

TREO stands for Total rare earth. This abbreviation is used consistently in all subsequent tables and figures.

### 3.2 Comparison of element contents in oolong tea from different producing regions

Elemental analysis of tea samples from the three major producing regions (Fujian, Guangdong, and Taiwan) revealed significant regional differences (*p* < 0.001), with specific results presented in Table 2 and Supplementary Figure 2. Oolong tea produced in Fujian Province exhibited the highest level of elemental enrichment. The average Pb content of Fujian tea was 0.524 ± 0.310 mg/kg, which was significantly higher than that of samples from Guangdong (0.266 ± 0.200 mg/kg) and Taiwan (0.142 ± 0.130 mg/kg). A consistent trend was observed for Al content: the Al content in Fujian tea was 1275.031 ± 2269.000 mg/kg, whereas those in Guangdong and Taiwan teas were 634.868 and 711.000 mg/kg, respectively. Similarly, the content of TREO in Fujian-produced tea (2.988 ± 3.000 mg/kg) was significantly higher than that in samples from Guangdong (1.178 ± 1.200 mg/kg) and Taiwan (0.385 ± 0.300 mg/kg). In contrast, no statistically significant difference in Cd content was detected among the three producing regions (*p* = 0.131).

**Table 2:** Comparison of leached toxic elements and rare earth in oolong tea leaves from different regions.

Element	Region			*p-*value
	Fujian	Guangdong	Taiwan	
Pb (mg/kg)	0.524 ± 0.310	0.266 ± 0.200	0.142 ± 0.130	<0.001
Cd (mg/kg)	0.031 ± 0.018	0.025 ± 0.016	0.017 ± 0.018	0.131
Al (mg/kg)	1275.031 ± 2269.000	634.868 ± 1536.000	711.000 ± 327.000	<0.001
Sc (mg/kg)	0.048 ± 0.034	0.026 ± 0.016	0.017 ± 0.011	<0.001
Y (mg/kg)	0.436 ± 0.580	0.158 ± 0.170	0.050 ± 0.012	<0.001
La (mg/kg)	0.407 ± 0.560	0.147 ± 0.180	0.061 ± 0.045	<0.001
Ce (mg/kg)	0.829 ± 0.820	0.369 ± 0.340	0.083 ± 0.039	<0.001
Pr (mg/kg)	0.080 ± 0.090	0.028 ± 0.030	0.012 ± 0.008	<0.001
Nd (mg/kg)	0.330 ± 0.350	0.116 ± 0.120	0.052 ± 0.020	<0.001
Sm (mg/kg)	0.059 ± 0.060	0.021 ± 0.020	0.010 ± 0.005	<0.001
Eu (mg/kg)	0.013 ± 0.010	0.005 ± 0.004	0.003 ± 0.001	<0.001
Gd (mg/kg)	0.061 ± 0.060	0.021 ± 0.020	0.010 ± 0.005	<0.001
Tb (mg/kg)	0.010 ± 0.010	0.003 ± 0.003	0.002 ± 0.001	<0.001
Dy (mg/kg)	0.062 ± 0.070	0.023 ± 0.030	0.007 ± 0.003	<0.001
Ho (mg/kg)	0.013 ± 0.010	0.005 ± 0.005	0.002 ± 0.001	<0.001
Er (mg/kg)	0.045 ± 0.060	0.017 ± 0.020	0.004 ± 0.002	<0.001
Tm (mg/kg)	0.007 ± 0.010	0.003 ± 0.003	0.002 ± 0.001	<0.001
Yb (mg/kg)	0.054 ± 0.070	0.023 ± 0.030	0.003 ± 0.001	<0.001
Lu (mg/kg)	0.009 ± 0.010	0.004 ± 0.004	0.002 ± 0.001	<0.001
TREO (mg/kg)	2.988 ± 3.000	1.178 ± 1.200	0.385 ± 0.300	<0.001

### 3.3 Leaching behavior under different temperatures and brewing durations

Brewing temperature had a significant impact on the leaching behavior of most elements (Table 3; Supplementary Figure 1). However, when the brewing time was fixed, temperature (90°C vs. 100°C) showed no significant effect on the leaching amounts of Pb, Al, Cu, Mn, and Ni (*p* > 0.05). Across all brewing durations, the leaching amount of Cd at 100°C was significantly lower than that at 90°C. The effect of temperature on REEs leaching exhibited element-specific characteristics (Table 4; Supplementary Figure 2). The leaching of Sc increased with rising temperature, while the leaching amount of Y at 100°C was generally lower than that at 90°C. No significant differences in the leaching amounts of other REEs were observed between the two temperatures.

**Table 3:** Comparison of toxic element leaching amounts at different brewing temperatures.

Temperature	Time	Toxic element content					
		Pb (μg/L)	Cd (μg/L)	Al (mg/L)	Cu (mg/L)	Mn (mg/L)	Ni (mg/L)
90°C	5 s	0.179	0.064^**^	0.535	0.006	0.437	0.004
	1 min	0.282	0.061^**^	0.806	0.008	0.677	0.006
	5 min	0.457	0.069	1.635	0.015	1.193	0.012
	10 min	0.504	0.08^***^	2.216	0.018	1.541	0.016
	30 min	0.578	0.075^***^	3.294	0.023	2.267	0.021
	120 min	0.662	0.081^***^	4.713	0.036	3.538	0.032
	*P*-value	<0.001	<0.001	<0.001	<0.001	<0.001	<0.001
100°C	5 s	0.143	0.046^**^	0.401	0.005	0.409	0.004
	1 min	0.296	0.051^**^	0.849	0.008	0.804	0.008
	5 min	0.572	0.062	1.809	0.012	1.375	0.013
	10 min	0.658	0.061^***^	2.45	0.015	1.737	0.017
	30 min	0.572	0.061^***^	3.634	0.021	2.653	0.023
	120 min	0.709	0.06^***^	4.361	0.03	3.369	0.029
	*P*-value	<0.001	<0.001	<0.001	<0.001	<0.001	<0.001

*P < 0.05, **P < 0.01, and ***P < 0.001 in comparison of the same brewing time.

**Table 4:** Comparison of rare earth oxide leaching amounts at different brewing temperatures (Unit: μg/L).

Temperature	Brewing time	Rare earth content																
		Sc	Y	La	Ce	Pr	Nd	Sm	Eu	Gd	Tb	Dy	Ho	Er	Tm	Yb	Lu	TREO
90°C	5 s	0.316	0.102**	0.038	0.086	0.009	0.038	0.007	0.009	0.009	0.001	0.011	0.003	0.009*	0.001	0.011	0.002	0.921
	1 min	0.322	0.11	0.046	0.114	0.01	0.043	0.009	0.009	0.01	0.002	0.011	0.003	0.01	0.002	0.014	0.002	0.996
	5 min	0.342**	0.162	0.066	0.174	0.015	0.071	0.014	0.009	0.016	0.003	0.017	0.004	0.015	0.003	0.019	0.003	1.213
	10 min	0.358*	0.212	0.08	0.193	0.018	0.075	0.015	0.009	0.018	0.003	0.023	0.006	0.02	0.004	0.027	0.005	1.445
	30 min	0.391**	0.249	0.099	0.237	0.021	0.099	0.019	0.009	0.022	0.004	0.026	0.006	0.024	0.004	0.03	0.005	1.798
	120 min	0.444	0.305	0.113	0.311	0.027	0.117	0.024	0.011	0.028	0.005	0.032	0.008	0.026	0.005	0.034	0.006	1.998
	*P*-value	<0.001	<0.001	<0.001	<0.001	<0.001	<0.001	<0.001	0.299	<0.001	<0.001	<0.001	<0.001	<0.001	<0.001	<0.001	<0.001	<0.001
100°C	5 s	0.321	0.082**	0.033	0.076	0.007	0.031	0.006	0.009	0.007	0.001	0.008	0.002	0.007*	0.001	0.008	0.001	0.865
	1 min	0.332	0.099	0.051	0.111	0.01	0.047	0.009	0.009	0.01	0.001	0.011	0.003	0.009	0.002	0.012	0.002	1.015
	5 min	0.365**	0.133	0.067	0.164	0.015	0.064	0.012	0.009	0.013	0.002	0.016	0.004	0.012	0.002	0.017	0.003	1.254
	10 min	0.379*	0.15	0.077	0.195	0.017	0.07	0.013	0.009	0.016	0.002	0.019	0.004	0.015	0.003	0.018	0.003	1.392
	30 min	0.417**	0.239	0.113	0.275	0.023	0.102	0.02	0.01	0.022	0.003	0.025	0.006	0.023	0.004	0.027	0.005	1.788
	120 min	0.453	0.281	0.118	0.299	0.027	0.12	0.025	0.01	0.028	0.004	0.031	0.008	0.027	0.005	0.034	0.005	2.036
	*P*-value	<0.001	<0.001	<0.001	<0.001	<0.001	<0.001	<0.001	0.938	<0.001	<0.001	<0.001	<0.001	<0.001	<0.001	<0.001	<0.001	<0.001

*P < 0.05, **P < 0.01 and ***P < 0.001 in comparison of the same brewing time.

Regardless of the brewing temperature (90°C or 100°C), prolonging the brewing time significantly increased the leaching amounts of all toxic elements and REEs (*p* < 0.001). As shown in Table 3, the leaching amounts of toxic elements (Pb, Cd, Al, Cu, Mn, Ni) exhibited a continuous increasing trend from 5 s to 2 h. For instance, at 90°C, the leaching amount of Pb increased from 0.179 μg/L at 5 s to 0.662 μg/L at 2 h. The leaching pattern of REEs was similar (Table 4). The leaching rate of TREO reached a peak in the initial brewing stage (5–10 min), after which the growth rate slowed down and gradually stabilized. The leaching rate of Sc remained at a relatively high level under both temperatures.

### 3.4 Analysis of leaching rate

**Figure 1:**
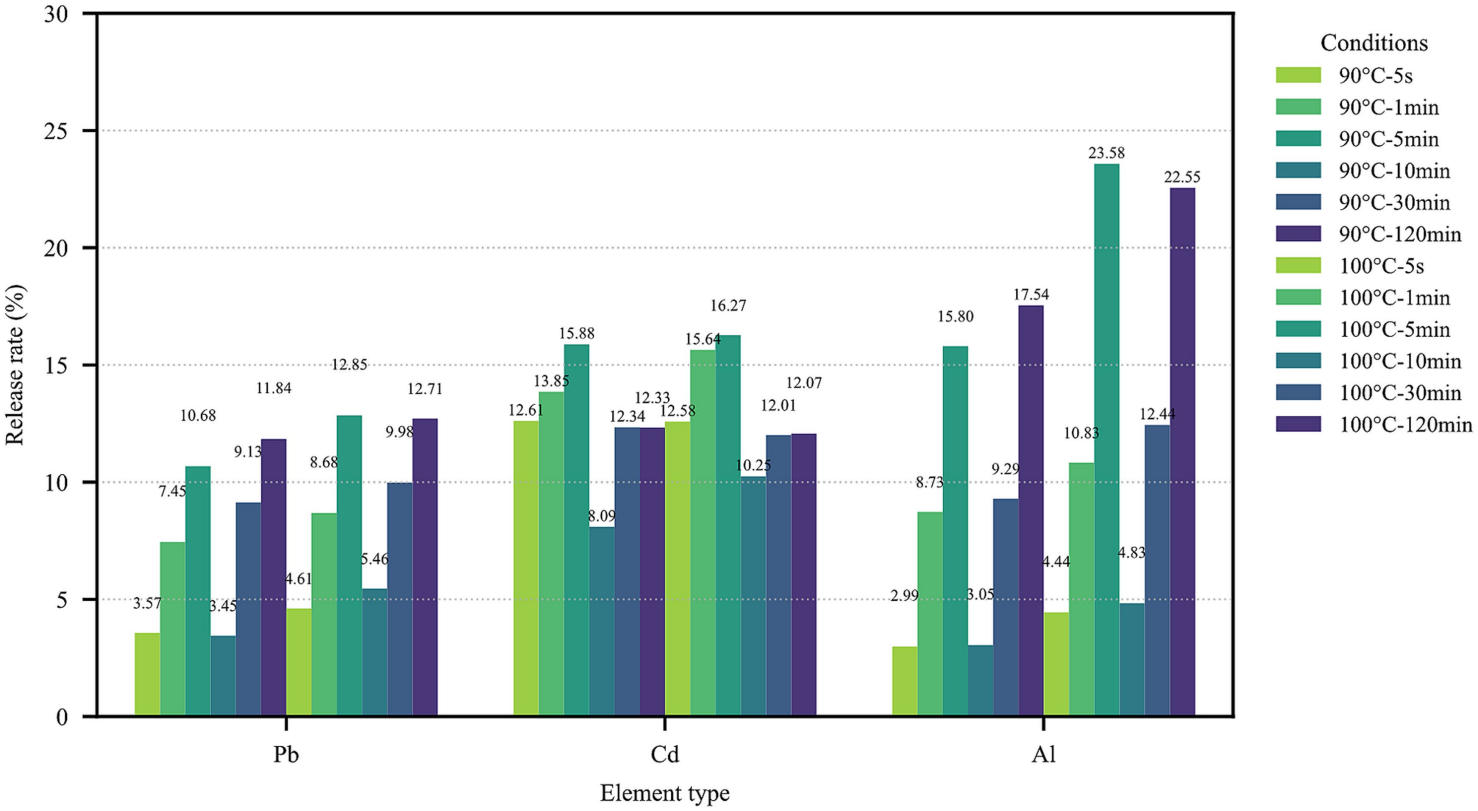
Comparison of toxic element release rates under different conditions.

**Figure 2:**
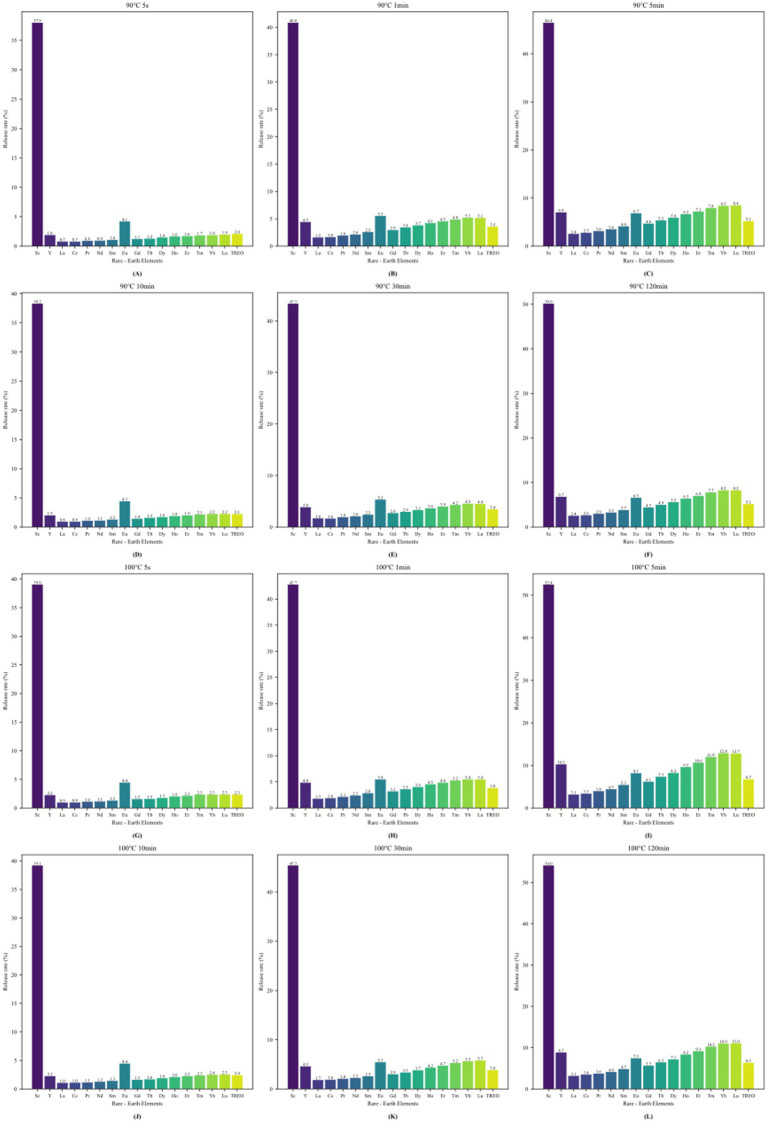
Comparison of rare earth oxide precipitation rates under different conditions.

Analysis of the leaching rate further confirmed the aforementioned findings (Figures 1, 2). The overall leaching rate at the higher temperature (100°C) was higher than that at 90°C. Most elements leached most rapidly within the first 5–10 min of brewing, after which the leaching kinetic curves gradually flattened. Notably, although the absolute leaching amount of Cd at 100°C was relatively low, its leaching dynamics were similar to those of other elements, increasing with the extension of time. The leaching rates of REEs at 100°C were also significantly higher than those at 90°C, especially within the critical time window of 5–10 min.

## 4 Discussion

This study systematically examined how infusion temperature and time influence the leaching of toxic elements and REEs from oolong tea. The release of Pb, Al, Cu, Mn, and Ni increased steadily as the brewing time was extended, peaking at the 120-min mark. This trend is consistent with earlier findings that extended steeping enhances heavy metal migration (23, 24), in contrast, cadmium showed reduced release at 100°C, while scandium exhibited greater release, suggesting element-specific behaviors under thermal conditions. Such atypical patterns are partly explained by strong binding of Cd to polyphenols or proteins, which can reduce its solubility at higher temperatures (25–28). These results emphasize the importance of both elemental speciation and organic matrix composition in shaping leaching patterns.

This study found that varietal and regional effects were also evident. Tieguanyin contained the highest Pb, Al and total REEs, while Dahongpao, Dancong and Narcissus showed lower concentrations. Regionally, Fujian teas exhibited greater enrichment of Pb, Al and REEs than Guangdong and Taiwan samples, in line with evidence that soil geochemistry and cultivation practices significantly influence contaminant accumulation (29, 30). Such differences highlight the need to account for terroir and production conditions when assessing health risks associated with tea consumption.

Kinetic analysis indicated that most elements leached most rapidly within the first 5–10 min, followed by slower increases and eventual plateauing. This early-phase acceleration agrees with reports that initial brewing dominates elemental transfer (23, 24). The divergent responses of Cd and Sc further underscore the need to treat individual elements separately when assessing infusion chemistry.

The experimental design do not fully replicate consumer practices of repeated short infusions. Furthermore, the present measurements reflect leached concentrations but not gastrointestinal bioaccessibility, which may lead to differences between leaching potential and actual absorbed dose.

The limitation of this study is its exclusion of sequential infusion models and *in vitro* digestion systems. Therefore, future studies should expand to include these, with a focus on clarifying the chemical speciation of Cd, Sc and other elements that exhibit non-linear responses to temperature. Broader geographic and temporal sampling will also be important for improving generalizability. Despite these limitations, this study provides detailed kinetic evidence on toxic element and REE release, demonstrating how varietal, regional and infusion factors collectively shape contaminant migration from tea leaves to infusion.

## 5 Conclusion

This study revealed distinct varietal and regional differences in elemental enrichment. Tieguanyin contained the highest concentrations of Pb, Al and REEs, and teas from Fujian showed significantly higher levels than those from Guangdong and Taiwan. Infusion experiments demonstrated that Pb, Al, Cu, Mn, and Ni increased with steeping time, with the most rapid release occurring within the first 5–10 min. Cd showed lower release at 100°C than at 90°C, whereas Sc displayed higher release at 100°C.

These findings demonstrate that varietal traits, geographic origin and infusion kinetics jointly shape contaminant migration from tea leaves to infusions. While current national standards, including GB2762-2022 and GB5749-2022, define baseline limits, the variability observed here highlights the need for continued surveillance and possible refinement to ensure consumer safety.

## Data Availability

The original contributions presented in the study are included in the article/Supplementary material, further inquiries can be directed to the corresponding author.
