# Correction: Investigation of the impact of brewing parameters on toxic element and rare earth element contamination in oolong tea

**DOI:** 10.3389/fnut.2025.1744585

**Published:** 2025-11-27

**Authors:** Sixuan Wu, Ziming Peng, Yijun Ou, Florence Mhungu, Yanyan Wang, Yuhua Zhang, Lan Liu, Jiangbo Lei, Lili Huang, Rongfei Peng, Zhijun Bai, Weiwei Zhang

**Affiliations:** 1 Department of Public Health and Preventive Medicine, School of Medicine, Jinan University, Guangzhou, China; 2 Department of Foodborne Disease and Food Safety Risk Surveillance, Guangzhou Center for Disease Control and Prevention (Guangzhou Health Supervision Institute), Guangzhou, China; 3 Pingxiang Center for Disease Control and Prevention, Pingxiang, China; 4 School of Economics and Statistics, Guangzhou University, Guangzhou, China; 5 Horizon Health Network, Fredericton, NB, Canada; 6 School of Public Health, Guangdong Pharmaceutical University, Guangzhou, China; 7 Department of Quality Control, Guangzhou Center for Disease Control and Prevention (Guangzhou Health Supervision Institute), Guangzhou, China; 8 Department of Physical and Chemical Inspection, Guangzhou Center for Disease Control and Prevention (Guangzhou Health Supervision Institute), Guangzhou, China

**Keywords:** oolong tea, toxic elements, REEs, brewing conditions, ICP-MS, food safety

Author Weiwei Zhang was erroneously assigned to affiliation “Department of Public Health and Preventive Medicine, School of Medicine, Jinan University, Guangzhou, China”.

This affiliation has now been removed for author Weiwei Zhang.

Affiliation “Department of Foodborne Disease and Food Safety Risk Surveillance, Guangzhou Center for Disease Control and Prevention (Guangzhou Health Supervision Institute), Guangzhou, China” was omitted for the author Weiwei Zhang.

This affiliation has now been added for author Weiwei Zhang.

Affiliations 1 and 2 were numbered incorrectly.

Incorrect order:

“1Department of Foodborne Disease and Food Safety Risk Surveillance, Guangzhou Center for Disease Control and Prevention (Guangzhou Health Supervision Institute), Guangzhou, China

2Department of Public Health and Preventive Medicine, School of Medicine, Jinan University, Guangzhou, China”

The Correct order is:

“^1^Department of Public Health and Preventive Medicine, School of Medicine, Jinan University, Guangzhou, China

^2^Department of Foodborne Disease and Food Safety Risk Surveillance, Guangzhou Center for Disease Control and Prevention (Guangzhou Health Supervision Institute), Guangzhou, China.”

The original version of this article has been updated.

