# Supplementary figures and images for: Investigation of the impact of brewing parameters on toxic element and rare earth element contamination in oolong tea
Source: Front Nutr. 2025 Nov 11;12:1656046. doi: 10.3389/fnut.2025.1656046 (PMC12646019; doi:10.3389/fnut.2025.1656046)

**Toxic Metal Dissolution: 90°C vs 100°C (Log-Transformed for Clarity)**

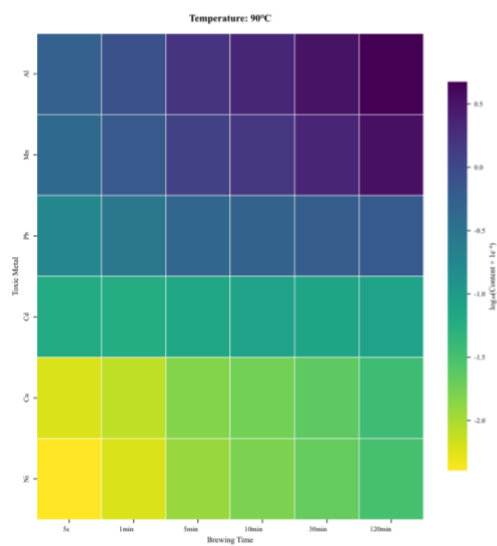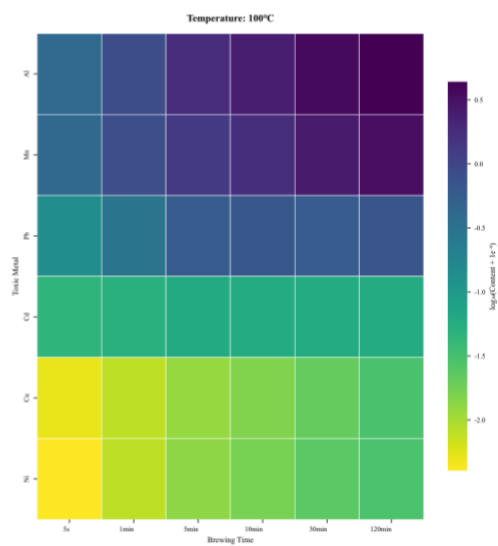

Supplement: Supplementary file 1 [file Image_1.pdf]

### Rare Earth Oxide Precipitation: 90°C vs 100°C (Log-Transformed for Clarity)

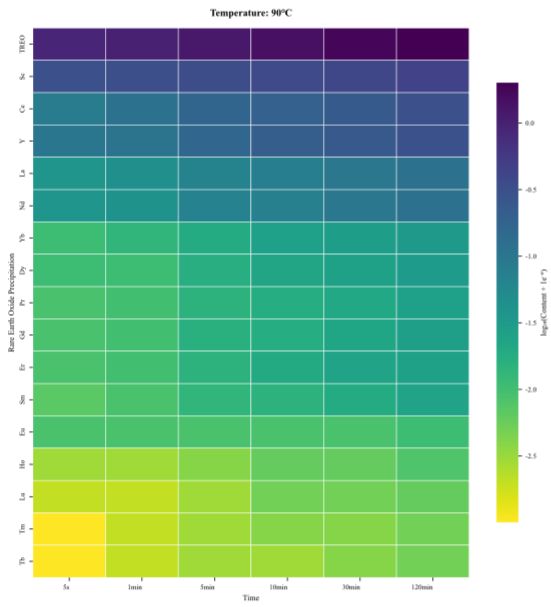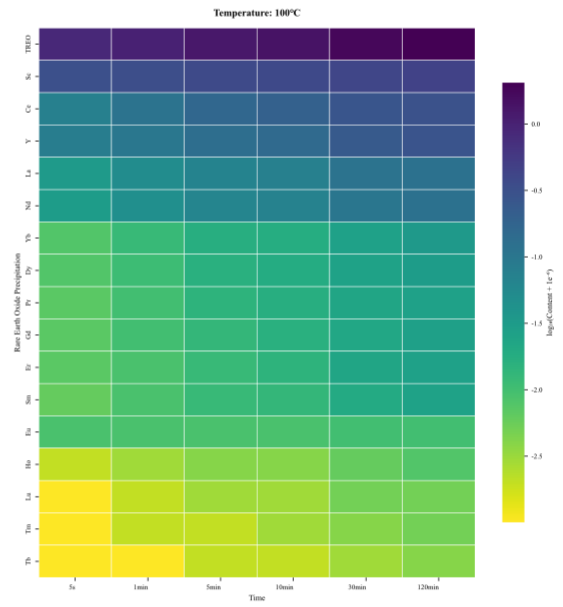

Supplement: Supplementary file 2 [file Image_2.pdf]
